# Pulmonary Embolism and Cardiovascular Care: Revolutionary Advances for the Decade Ahead

**DOI:** 10.14797/mdcvj.1398

**Published:** 2024-05-16

**Authors:** Thomas M. Loh, Alan B. Lumsden

**Affiliations:** 1 Houston Methodist DeBakey Heart & Vascular Center, Houston Methodist, Houston, Texas, US

**Keywords:** pulmonary embolism, pulmonary embolism response team (PERT), massive PE, deep vein thrombosis (DVT), venous thromboembolism (VTE), chronic thromboembolic pulmonary hypertension

## Abstract

Introductory overview for *Methodist DeBakey Cardiovascular Journal* Issue 20.3 on Pulmonary Embolism, written by the issues’ guest editors.

Viva la revolución! We are in the midst of an evolving transformation in the care of pulmonary embolism (PE). The 1990s saw the rise of primary percutaneous coronary intervention as the de-facto treatment modality for myocardial infarction (MI). Similarly, the 2010s saw the rise of mechanical thrombectomy as the preferred method of treating large vessel occlusions in stroke. These changes had been virtually unthinkable just a decade before. Two factors aided in these foundational changes in care: Better fundamental understanding of the pathologies and, more importantly, rapidly improving endovascular toolkits specifically designed for the target vascular beds.

Pulmonary embolism is now the “third horseman” of cardiovascular death, behind only MI and stroke. In this issue, we seek to provide a foundation of knowledge to serve as a springboard for exploring the rapidly changing care of pulmonary embolism—and new possibilities for reducing its impact.

As with many aspects of PE, timely diagnosis and management by a team of specialists is critical. Assessment by a Pulmonary Embolism Response Team (PERT) has proven significant, aided by automated detection and flagging of suspected PE in computed tomography pulmonary angiography imaging. In “New Diagnostic Tools for Pulmonary Embolism Detection,” Drs. Patrick Muck, Jacob Shapiro, and Adam Reichard describe the newest diagnostic tools using artificial intelligence to facilitate workflow with quick access to imaging and sharing real-time information to improve triage and diagnosis.

In “Intervention Versus Medical Management of Pulmonary Embolism,” I (Thomas Loh) review the most common diagnostic tools for assessing risk of PE, explore each broad risk category, and describe typical approaches for management. I have included an algorithm to clarify the decision-making process, focusing on right heart dysfunction as the cornerstone for triage for these patients, especially those facing the gravest challenges.

Our focus next turns to massive PE (MPE), a serious condition affecting the pulmonary arteries. In “From Trendelenburg to PERTs: Evolution in the Management of Massive Pulmonary Embolism,” Dr. Pavan Thangudu discusses the challenges of diagnosing, triaging, and treating MPE. He further explains the different classification approaches for PE presented by The American Heart Association and the European Society of Cardiology, which offers broader criteria for MPE, including cardiac arrest and obstructive shock.

Deep vein thrombosis (DVT) and especially pulmonary embolus both carry significant impending morbidity and mortality. While anticoagulation in the outpatient setting is the standard of care for patients with low risk, the choice of initial anticoagulation that allows for therapeutic adjustment or manipulation is crucial for intermediate- and high-risk patients, for whom revascularization may be necessary based on clinical deterioration. Dr. Joseph Naoum reviews the primary and longer-term considerations of anticoagulation management for these patients with PE and highlights special patient populations and risk-factor considerations in “Anticoagulation Management Post Pulmonary Embolism.”

Traditional treatment options for PE are fraught with bleeding risks and incomplete thrombus removal, clearly necessitating the development of innovative treatment strategies. While new interventional approaches offer promising potential, their rapid development and dearth of comparative clinical evidence pose challenges for selecting the optimal treatment for each patient. In “Catheter Interventions for Pulmonary Embolism: Mechanical Thrombectomy versus Thrombolytic,” Dr. Nicolas Mouawad summarizes currently published clinical data for both traditional and recent interventional approaches. Further, he describes studies still in progress that may soon prove critical for helping physicians make informed treatment choices and potentially driving needed guideline changes.

For patients who have developed a DVT and/or a PE and cannot tolerate anticoagulation, inferior vena cava (IVC) filters are a key consideration. In “Inferior Vena Cava Filters: An Overview,” Dr. Maham Rahimi and coauthors Paul Haddad, Jasmine Peng, and Madeline Drake discuss important factors and techniques that surgeons and interventionalists should be aware of and prepared to consider. This overview addresses the basics regarding the history of filters, indications for placement, the associated risks, and techniques for difficult removal.

Chronic thromboembolic pulmonary hypertension is a rare form of pulmonary hypertension in patients who have evidence of chronic thromboembolic occlusion of the pulmonary vasculature. Although surgical pulmonary thromboendarterectomy historically has been the treatment of choice, balloon pulmonary angioplasty has emerged as an additional treatment strategy for the 40% of patients who are deemed inoperable. This complementary strategy, alongside surgical pulmonary thromboendarterectomy, offers the opportunity for pulmonary revascularization in patients who have more distal disease, higher comorbidities, or residual obstruction following operative intervention. Drs. Zachary Steinberg, Lauren Carlozzi, and Huie Lin review the history of balloon pulmonary angioplasty, highlight its effectiveness, and discuss important complications and risk reduction strategies. They also emphasize the importance of centers with a multidisciplinary team of providers to manage the complexity of patients with chronic thromboembolic pulmonary hypertension.

Though PE continues to pose risks and challenges, new devices, current clinical studies, increased awareness, and efficient team approaches offer renewed hope for revolutionary advances in the decade ahead.

## Guest Editor Biography

The editors of the *Methodist DeBakey Cardiovascular Journal* express our appreciation to Dr. Thomas M. Loh and Dr. Alan B. Lumsden for their knowledge and insight in developing this issue on pulmonary embolism.

## Thomas M. Loh, MD

**Figure:**
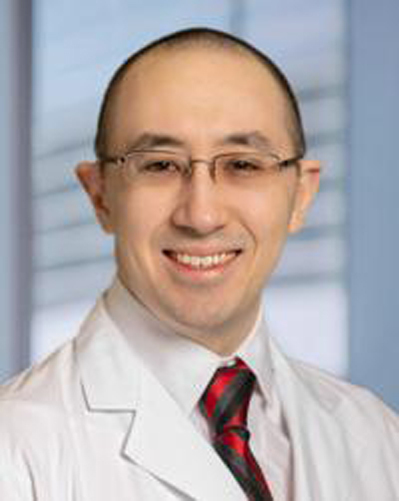


Dr. Loh joined the faculty of the Houston Methodist DeBakey Heart & Vascular Center at Houston Methodist in 2019. During the COVID-19 pandemic, he developed one of the largest and busiest pulmonary embolism programs in a community setting. He is committed not only to the treatment of patients but also to system/program development. This commitment has led him to provide support to more than a dozen other nascent pulmonary embolism programs across the United States.

Dr. Loh’s current focus is the Venous Thromboembolism (VTE) Center Of Excellence. He puts an emphasis not only on exceptional medical care but also on being responsible stewards of healthcare resources and sustainable systems.

## Alan B. Lumsden, MD

**Figure:**
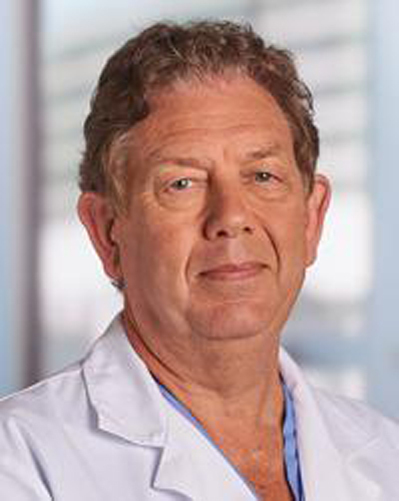


Dr. Lumsden’s academic career began in 1989 at Emory University in Atlanta, Georgia, where he completed his postdoctoral training and served as a Collaborative Scientist in the Division of Pathobiology and Immunology at the renowned Yerkes Primate Center. He remained at Emory for several years, rising to the rank of associate professor and chief of the Division of Vascular Surgery. In 2002, Dr. Lumsden joined the faculty at the Baylor College of Medicine in Houston, Texas, as professor and chief of the Division of Vascular Surgery and Endovascular Therapy. He assumed his current positions at Houston Methodist in 2008.

Dr. Lumsden has developed an international reputation as a leader in the field of endovascular surgery. He has clinical and research expertise in stent graft treatment of thoracic and abdominal aortic aneurysms, stenting and endarterectomy in carotid arterial disease, renovascular hypertension, aortoiliac occlusive disease, and mesenteric vascular and minimally invasive therapy in venous disease. Dr. Lumsden also conducts FDA-mandated training for surgeons nationwide in a carotid stenting simulator housed at the Houston Methodist DeBakey Heart & Vascular Center.

Dr. Lumsden’s research interests include developing novel minimally invasive methods of therapy and preventing restenosis. His research has been funded by the National Institutes of Health, and he has contributed more than 200 papers to the medical literature as well as numerous abstracts, books, and book chapters.

